# Grip and pinch strengths and its association with cardiometabolic risk in children and adolescents aged 6 to 17 years

**DOI:** 10.3389/fnut.2026.1763759

**Published:** 2026-02-17

**Authors:** Yiren Chen, Yiying Huang, Lijun Wu, Zijun Liao, Shaoli Li, Junting Liu, Xinnan Zong, Fangfang Chen

**Affiliations:** Department of Epidemiology, Capital Center for Children’s Health, Capital Medical University, Capital Institute of Pediatrics, Beijing, China

**Keywords:** adolescents, cardiometabolic risk, children, grip strength, pinch strength

## Abstract

**Background:**

To describe the absolute and body composition-normalized relative grip and pinch strength status in children and adolescents, to analyze the associations between strength indices and cardiometabolic risk.

**Methods:**

The baseline survey from 2022 to 2023 conducted by the Beijing Children and Adolescents Health Cohort. Children aged 6–17 years old in Beijing were involved (*N* = 3,252). Health examination included height, body composition, grip and pinch strengths, blood pressure, triglycerides (TG), low-density lipoprotein cholesterol (LDL-c), etc. Clustered metabolic syndrome composite score (MetScore) was calculated. Grip and pinch strengths were normalized by muscle mass percentage (MMP), body weight, BMI and muscle mass as strength indices, recorded as Grip-to-MMP ratio, etc. The receiver operating characteristic (ROC) curves were generated for age- and sex-specific muscle strength indices predicting abnormal cardiometabolic parameters.

**Results:**

3,252 children and adolescents were included. Both grip and pinch strength increased with age, with boys stronger than girls. Absolute grip strength and Grip-to-MMP ratio were positively associated with the risks of developing low HDL-c, high LDL-c, HBP, and a high MetScore. In contrast, the Grip-to-WT ratio was negatively associated with the risks of high TG, low HDL-c, high LDL-c, HBP, and a high MetScore, while being positively associated with the risk of IFG. Similar patterns were observed for pinch strength and its normalized indices. The Pinch-to-WT ratio and Pinch-to-BMI ratio showed protective, inverse associations with the risks of high TG, low HDL -c, high LDL-c, HBP, and a high MetScore. ROC analysis identified the Grip-to-WT ratio as the strongest predictor of cardiometabolic risk among all indices evaluated.

**Conclusion:**

Grip and pinch strengths seemed associated with cardiometabolic risk in children. The Grip/Pinch-to-WT ratio was the best predictor of cardiometabolic risk than all the other strength indices. Testing grip strength might help prevent cardiovascular disease early.

## Introduction

Childhood and adolescence are the most critical stages of physical and mental development in an individual’s entire life cycle (1), and muscle strength, as the basis of exercise, is an important dimension reflecting an individual’s level of health (2, 3). Grip strength, as the maximum force generated by simultaneous contraction of the muscles of the palm and forehand, is closely related to total muscle strength and therefore is often used as a quick indicator of the overall muscle strength of the human body tool (4). Low relative grip strength was associated with a higher risk of cardiometabolic disease (5). In addition, pinch strength is another manifestation of muscle strength: the strength generated by pinching the thumb and index fingers together without the use of the palm of the hand (6). Higher cumulative muscle strength was associated with lower risk of diabetes and better cardiometabolic health (7). Higher muscle strength might help reduce cardiometabolic risk (8, 9). Measuring grip strength and pinch strength might help predict cardiometabolic health risk (10).

Cardiometabolic risk factors include weight status, triglycerides (TG), low-density lipoprotein cholesterol (LDL-C), high-density lipoprotein cholesterol (HDL-C), fasting blood glucose (FPG), systolic blood pressure (SBP) and diastolic blood pressure (DBP) (11). As cardiometabolic risk is closely associated with obesity, its prevalence and severity are expected to rise alongside the increasing obesity rates in Chinese children (12). And chronically high levels of cardiometabolic risk indicators predict a high risk of metabolic abnormalities in adolescence and adulthood (13). Therefore, monitoring cardiometabolic risk indicators is essential in public health policy, especially in pediatric populations (14). In child and adolescent populations, muscle strength was associated with a lower risk related to obesity and a lower risk related to aggregated cardiometabolic variables, but associations with blood pressure, lipids, and blood glucose have not been consistent (15, 16).

Grip strength and pinch strength are strongly correlated with an individual’s sex, age, and weight status, including body weight (WT), body mass index (BMI) and muscle mass (MM) (17, 18). Thus, it is necessary to analyze the association between grip strength and pinch strength standardized by body weight status and cardiometabolic risk, which could help to distinguish child with abnormal cardiometabolic parameter more accurately (19, 20), and would greatly simplify the process of screening children for health problems.

Although previous studies have showed a correlation between muscle strength and cardiometabolic risk, there has been a lack of studies that take into account the level of childhood development. This study aims to describe the absolute and body composition-normalized relative grip and pinch strength status in children and adolescents, to analyze the associations between strength indices and cardiometabolic risk.

## Methods

This report followed the Strengthening the Reporting of Observational Studies in Epidemiology (STROBE) reporting guidelines for cross-sectional studies (21).

### Study design and population

The data for this study came from the baseline survey from 2022 to 2023 conducted by the Beijing Children and Adolescents Health Cohort, which was a prospective cohort study design. We obtained written informed consent from participants/guardians. The studies involving human participants were reviewed and approved by the ethics committee of Capital Institute of Pediatrics, Beijing, China (SHERLL2022043). This study only extracted data from children and adolescents aged 6 to 17 years to ensure the accuracy of grip and pinch strength measurements. A total of 3,252 healthy children and adolescents aged 6 to 17 years participated in this survey, with the exception of those who could not participate in the physical examination due to trauma and physical discomfort. Exclusion criteria: (1) having a serious chronic disease or major health problem; (2) have a severe developmental or cognitive impairment; (3) having arm injuries or any other condition that could impact grip and pinch strength.

### Exposures

To address potential sources of bias, all assessments were conducted by trained data collectors, most of whom were nurses and doctors.

### Basic information

The children’s sex and age were obtained through a structured questionnaire.

### Height and body composition measurements

The mechanical stadiometer (Harpenden Portable Stadiometer, UK) was used in height measurements. The four-electrode eight-point contact bioresistive resistance method (Seehigher BAS-H, Beijing, China) was used in body composition measurements (22, 23). The height, WT and MM were recorded accurately to 0.1 cm or 0.1 kg, and then BMI was calculated as body weight (kg)/height^2^ (m^2^), and muscle mass percentage (MMP) was calculated as MM (kg)/weight (kg).

### Cardiometabolic risk indicators

Blood pressure was measured using an oscillometric sphygmomanometer (HBP-1300, Omron, Japan) in the brachial artery of the right upper arm, the total of 3 measurements were taken to record SBP and DBP in mmHg, and the average of the last 2 measurements was taken as the analyzed value.

Venous blood (5 mL) was collected from the participants after 12 h of fasting, and FPG was measured by the glucokinase method, HDL-c and LDL-c were measured by the direct method, TG was measured by the GPO-PAP method, all in mmol·L^−1^, and the instruments used were Siemens Automatic Biochemistry Analyzer (ADVIA 2400, Siemens, Germany).

### Grip strength and pinch strength measurements

Grip strength measurement: The participants’ maximal isometric grip strength was measured using the Hand Dynamometer (JAMAR^®^ Hydraulic Hand Dynamometer, Sammons Preston, USA), the participants were instructed to grip and hold for at least 5 s and 2 measurements in each hand.

Pinch strength was measured using the digital pinch gauge (Baseline^®^ pinch gauge, USA). The participants were instructed to squeeze with maximum strength and hold for at least 5 s and 3 measurements in each hand.

During the grip and pinch strength tests, the participants verbally motivated (24), and measurements were taken alternately with the right and left hands. The maximum grip and pinch strengths of both hands was recorded in kilograms (kg) to the nearest 0.1 kg. The specific operation implementation can refer to the standardized measurement method for grip strength (25).

### Outcome measures

#### Abnormal cardiometabolic parameter

High blood pressure (HBP) in children and adolescents was diagnosed according to the “Screening threshold for high blood pressure in children and adolescents aged 7–18 years” (26) and “Development of blood pressure reference standards for Chinese children and adolescents” (27).

Impaired fasting glucose (IFG) was evaluated using the American Diabetes Association’s recommended criteria for the diagnosis and classification of diabetes mellitus (28), and a FPG ≥ 5.6 mmol·L^−1^ was considered to indicate IFG.

Dyslipidemia was classified as follows (29): high TG: 9 years old and below ≥1.12 mmol·L^−1^, 10 years old and above, TG ≥ 1.46 mmol·L^−1^; low HDL-c: HDL-c < 1.03 mmol·L^−1^; and high LDL-c: LDL-c ≥ 3.36 mmol·L^−1^.

According to the International Diabetes Federation standard cutoff values (30), z scores were calculated for each of the following indicators according to age and sex: a clustered MetScore was constructed as a sum of the z scores: MetScore = (zBMI+ zFPG + zlgTG – zHDL- c + zSBP + zDBP)/6. The high MetScore was defined as an age- and sex-specific MetScore z score 1 or greater, which indicating increased cardiometabolic risk.

#### Grip and pinch strength indices

The maximum grip and pinch strengths of both hands were recorded as Grip strength and Pinch strength, and then Grip strength and Pinch strength normalized by body weight status (including WT, BMI, MM and MMP) were calculated and recorded as Grip-to-WT ratio, Grip-to-BMI ratio, Grip-to-MM ratio, Grip-to-MMP ratio, Pinch-to-WT ratio, Pinch-to-BMI ratio, Pinch-to-MM ratio and Pinch-to-MMP ratio.

### Statistical analyses

Data entry was performed using Epi Data 3.0 for two-person double entry. The consistency, logic, and boundary values of the data entered twice were checked by the data manager, and incorrect records were corrected according to the checking results and the questionnaire to ensure the accuracy of the data.

Smooth curves were fitted to the age-dependent changes in grip strength and pinch strength of children and adolescents aged 6 to 17 years. The means ± standard deviations (x ± s) were used to describe the means of all the quantitative indicators in the study that conformed to a normal distribution; the means of the indicators with a skewed distribution were described using the median [interquartile spacing] with M [IQR], and the number of people (percentage, %) was used to describe the distribution of each group for all the quantitative variables. Differences between sex were analyzed using *t* tests, *rank sum* tests, and *chi*-square tests. Associations between grip strength indices and cardiometabolic indices were analyzed using linear and Logistic regressions, and the receiver operating characteristic (ROC) curves were generated for age- and sex-specific muscle strength indices predicting abnormal cardiometabolic parameters, and their respective areas under the curve (AUCs) were compared. Analyses were performed using R Version 4.0.3^®^ (R Foundation for Statistical Computing, Vienna, Austria), the Statistical Package for Social Sciences (SPSS) software, version 26.0^®^ (IBM Corp., Armonk, NY, USA) and Med Calc software version 22.0^®^ (Med Calc software Ltd., Ostend, Belgium), and statistical significance was defined as a two-sided *p* < 0.05.

## Results

A total of 3,252 children and adolescents aged 6–17 years with complete grip and pinch strengths, body composition and cardiometabolic risk indicators were included in the final analysis.

Changes in grip strength and pinch strength with age according to sex are shown in Supplementary Figure S1. The grip and pinch strengths of both boys and girls tended to increase with age, and the grip and pinch strength of boys was greater than that of girls at all ages (*p* < 0.05, Supplementary Table S1). However, prior to puberty, the difference in grip strength and pinching strength between boys and girls decreased, while the difference increased during puberty (12 years and older).

Differences in the levels of body composition and cardiometabolic risk indicators between boys and girls are shown in Supplementary Table S2. The body composition indicators of boys were greater than those of girls, and the TG, HDL-c and SBP of boys were lower than those of girls; however, the FPG levels were greater than those of girls (*p* < 0.05), and the remaining cardiometabolic risk indicators were not significantly different between male and female students. The incidence of elevated blood pressure, low HDL-c and IFG were greater in boys than in girls (*p* < 0.05). The results of linear and Logistic regression analyses of grip and pinch strengths and cardiometabolic risk indices after adjusting for age, sex, annual family income, children’s dietary situation and children’s physical activity duration are shown in Tables 1, 2. Grip strength and Grip-to-MMP were positively correlated with TG, LDL-c, SBP, DBP and the MetScore, and were negatively correlated with HDL-c (*p* < 0.05). Grip -to-MMP ratio was positively correlated with FPG (*p* < 0.05). Grip-to-WT ratio, Grip -to-BMI ratio and Grip-to-MM ratio were negatively correlated with TG, FPG, SBP, DBP and MetScore, and were positively correlated with HDL-c (*p* < 0.05). Grip-to-WT ratio and Grip-to-BMI ratio were negatively correlated with LDL-c (*p* < 0.05). Pinch strength and Pinch-to-MMP were positively correlated with TG, FPG, SBP, DBP and the MetScore, and were negatively correlated with HDL-c (*p* < 0.05). Pinch-to-MMP ratio was positively correlated with LDL-c (*p* < 0.05). Pinch-to-WT ratio and Pinch-to-BMI ratio were negatively correlated with TG, SBP, DBP and MetScore, and were positively correlated with HDL-c (*p* < 0.05).

**Table 1:** Adjusted association between grip strength indices and cardiometabolic risks among participants of the study (*n* = 3,252).

Cardiometabolic variables^#^	Grip strength	Grip-to-WT ratio	Grip-to-BMI ratio	Grip-to-MM ratio	Grip-to-MMP ratio
	*β* (95%CI)	*β* (95%CI)	*β* (95%CI)	*β* (95%CI)	*β* (95%CI)
TG (mmol·L^−1^)	**0.003 (0.001, 0.006)**	**−1.104 (−1.236, −0.971)**	**−0.335 (−0.384, −0.286)**	**−0.439 (−0.567, −0.311)**	**0.009 (0.008, 0.011)**
HDL-c (mmol·L^−1^)	**−0.008 (−0.010, −0.006)**	**0.675 (0.581, 0.770)**	**0.167 (0.132, 0.202)**	**0.231 (0.140, 0.321)**	**−0.009 (−0.010, −0.008)**
LDL-c (mmol·L^−1^)	**0.006 (0.002, 0.010)**	**−1.184. (−1.409, −0.960)**	**−0.428 (−0.510, −0.346)**	−0.179 (−0.393, 0.034)	**0.013 (0.011, 0.015)**
FPG (mmol·L^−1^)	0.002 (−0.002, 0.005)	**−0.462 (−0.638, −0.286)**	**−0.103 (−0.168, −0.038)**	**−0.262 (−0.427, −0.097)**	**0.004 (0.002, 0.005)**
SBP (mmHg)	**0.448 (0.393, 0.503)**	**−24.167 (−27.320, −21.013)**	**−4.740 (−5.924, −3.557)**	**−7.180 (−10.218, −4.142)**	**0.412 (0.380, 0.444)**
DBP (mmHg)	**0.041 (0.002, 0.079)**	**−12.963 (−15.121, −10.805)**	**−3.537 (−4.334, −2.739)**	**−4.409 (−6.463, −2.356)**	**0.113 (0.089, 0.136)**
MetScore	**0.345 (0.237, 0.453)**	**−2.760 (−2.944, −1.319)**	**−0.771 (−0.842, −0.701)**	**−0.809 (−1.000, −0.618)**	**0.032 (0.030, 0.034)**

Abnormal cardiometabolic parameters^##^	Grip strength	Grip-to-WT ratio^*^	Grip-to-BMI ratio	Grip-to-MM ratio	Grip-to-MMP ratio
	OR (95%CI)	OR (95%CI)	OR (95%CI)	OR (95%CI)	OR (95%CI)
High TG	1.012 (0.996, 1.028)	**0.542 (0.486, 0.604)**	**0.131 (0.087, 0.198)**	**0.091 (0.036, 0.228)**	**1.039 (1.029, 1.049)**
Low HDL-c	**1.053 (1.035, 1.070)**	**0.612 (0.545, 0.687)**	**0.291 (0.194, 0.436)**	**0.185 (0.067, 0.512)**	**1.056 (1.045, 1.067)**
High LDL-c	**1.019 (1.002, 1.035)**	**0.709 (0.641, 0.784)**	**0.260 (0.177, 0.384)**	0.703 (0.291, 1.702)	**1.036 (1.027, 1.046)**
IFG	1.009 (0.986, 1.032)	**0.842 (0.729, 0.974)**	0.757 (0.460, 1.247)	0.451 (0.115, 1.775)	**1.014 (1.001, 1.028)**
HBP	**1.034 (1.018, 1.050)**	**0.523 (0.469, 0.583)**	**0.117 (0.078, 0.176)**	**0.099 (0.040, 0.248)**	**1.054 (1.044, 1.064)**
High MetScore	**1.058 (1.040, 1.077)**	**0.270 (0.231, 0.315)**	**0.027 (0.016, 0.047)**	**0.017 (0.006, 0.049)**	**1.103 (1.089, 1.117)**

^#^Linear regression analysis adjusted by age, sex, annual household income, dairy products, vegetable, fruits, fried food, sugared beverage and daily exercise duration. ^##^ Logistic regression analysis adjusted by age, sex, annual household income, dairy products, vegetable, fruits, fried food, sugared beverage and daily exercise duration. ^*^This Grip-to-WT ratio is the converted value obtained by multiplying the Grip-to-WT ratio value by 10 (i.e., expanding it to 10 times the original value).

Those highlighted in bold indicate statistical significance (bilateral *p* < 0.05, *p* values were from linear and logistics regression analysis).

MetScore = (zBMI+ zFPG + zlgTG - zHDL-c + zSBP+ zDBP)/6. The grip strength normalized by body weight status (including WT, BMI, MM, and MMP) were calculated and recorded as Grip-to-WT ratio, Grip-to-BMI ratio, Grip-to-MM ratio and Grip-to-MMP ratio.

BMI, body mass index; DBP, diastolic blood pressure; FPG, fasting blood glucose; HBP, high blood pressure; HDL-c, high-density lipoprotein cholesterol; IFG, Impaired fasting glucose; LDL, low-density lipoprotein cholesterol; MetScore, clustered metabolic syndrome composite score; MM, muscle mass; MMP, muscle mass percentage; SBP, systolic blood pressure; TG, triglycerides; WT, body weight.

**Table 2:** Adjusted association between pinch strength indices and cardiometabolic risks among participants of the study (*n* = 3,252).

Cardiometabolic variables^#^	Pinch strength	Pinch-to-WT ratio	Pinch-to-BMI ratio	Pinch-to-MM ratio	Pinch-to-MMP ratio
	*β* (95%CI)	*β* (95%CI)	*β* (95%CI)	*β* (95%CI)	*β* (95%CI)
TG (mmol·L^−1^)	**0.008 (0.003, 0.013)**	**−0.457 (−0.648, −0.265)**	**−0.224 (−0.318, −0.130)**	−0.052 (−0.198, 0.094)	**0.015 (0.012, 0.018)**
HDL-c (mmol·L^−1^)	**−0.009 (−0.012, −0.005)**	**0.371 (0.236, 0.506)**	**0.154 (0.088, 0.220)**	0.095 (−0.008, 0.198)	**−0.011 (−0.014, −0.009)**
LDL-c (mmol·L^−1^)	0.008 (−0.001, 0.016)	**−0.526 (−0.844, −0.208)**	**−0.343 (−0.498, −0.188)**	−0.003 (−0.245, 0.239)	**0.017 (0.012, 0.023)**
FPG (mmol·L^−1^)	**0.007 (0.001, 0.013)**	−0.068 (−0.315, 0.179)	0.001 (−0.120, 0.122)	0.039 (−0.149, 0.226)	**0.007 (0.003, 0.012)**
SBP (mmHg)	**0.564 (0.447, 0.680)**	**−10.271 (−14.807, −5.734)**	**−2.941 (−5.165, −0.717)**	−0.393 (−3.850, 3.064)	**0.581 (0.505, 0.657)**
DBP (mmHg)	**0.180 (0.100, 0.260)**	−2.580 (−5.654, 0.493)	−0.818 (−2.322, 0.686)	1.913 (−0.422, 4.248)	**0.229 (0.177, 0.282)**
MetScore	**0.032 (0.018, 0.031)**	**−1.266 (−1.551, −0.981)**	**−0.588 (−0.728, −0.449)**	−0.130 (−0.349, 0.089)	**0.046 (0.041, 0.050)**

Abnormal cardiometabolic parameters^##^	Pinch strength	Pinch-to-WT ratio^*^	Pinch-to-BMI ratio^**^	Pinch-to-MM ratio***	Pinch-to-MMP ratio
	OR (95%CI)	OR (95%CI)	OR (95%CI)	OR (95%CI)	OR (95%CI)
High TG	**1.041 (1.015**, **1.068)**	**0.676 (0.485**, **0.944)**	**0.817 (0.709**, **0.940)**	1.039 (0.956, 1.130)	**1.061 (1.039**, **1.083)**
Low HDL-c	**1.038 (1.009**, **1.067)**	**0.134 (0.084**, **0.214)**	**0.576 (0.484**, **0.687)**	**0.505 (0.343**, **0.742)**	**1.047 (1.028**, **1.067)**
High LDL-c	0.997 (0.962, 1.033)	**0.338 (0.232**, **0.491)**	**0.574 (0.488**, **0.674)**	0.890 (0.744, 1.064)	**1.025 (1.008**, **1.043)**
IFG	1.007 (0.952, 1.066)	**0.446 (0.250**, **0.796)**	0.886 (0.728, 1.077)	0.679 (0.408, 1.128)	1.019 (0.991, 1.048)
HBP	**1.028 (1.001**, **1.055)**	**0.148 (0.099**, **0.222)**	**0.481 (0.408**, **0.568)**	0.747 (0.559, 1.000)	**1.051 (1.031**, **1.072)**
High MetScore	**1.059 (1.032**, **1.087)**	**0.035 (0.021**, **0.059)**	**0.367 (0.302**, **0.447)**	0.808 (0.602, 1.083)	**1.133 (1.093**, **1.174)**

^#^Linear regression analysis adjusted by age, sex, annual household income, dairy products, vegetable, fruits, fried food, sugared beverage and daily exercise duration. ^##^Logistic regression analysis adjusted by age, sex, annual household income, dairy products, vegetable, fruits, fried food, sugared beverage and daily exercise duration. ^*^This Pinch-to-WT ratio is the converted value obtained by multiplying the Pinch-to-WT ratio value by 10 (i.e., expanding it to 10 times the original value). ^**^This Pinch-to-BMI ratio is the converted value obtained by multiplying the Pinch-to-BMI ratio value by 10 (i.e., expanding it to 10 times the original value). ^*^This Pinch-to-MM ratio is the converted value obtained by multiplying the Pinch-to-MM ratio value by 10 (i.e., expanding it to 10 times the original value).

Those highlighted in bold indicate statistical significance (bilateral *p* < 0.05, *p* values were from linear and logistics regression analysis).

MetScore = (zBMI+ zFPG + zlgTG - zHDL-c + zSBP+ zDBP)/6. The grip strength normalized by body weight status (including WT, BMI, MM and MMP) were calculated and recorded as Pinch-to-WT ratio, Pinch-to-BMI ratio, Pinch-to-MM ratio and Pinch-to-MMP ratio.

BMI, body mass index; DBP, diastolic blood pressure; FPG, fasting blood glucose; HBP, high blood pressure; HDL-c, high-density lipoprotein cholesterol; IFG, impaired fasting glucose; LDL, low-density lipoprotein cholesterol; MetScore, clustered metabolic syndrome composite score; MM, muscle mass; MMP, muscle mass percentage; SBP, systolic blood pressure; TG, triglycerides; WT, body weight.

Further logistic regression revealed that grip strength were positively associated with the risk of developing low HDL-c, high LDL-c, HBP and MetScore. Grip-to-WT ratio, Grip-to-BMI ratio and Grip-to-MM ratio were negatively associated with the risk of developing high TG, low HDL-c, HBP and MetScore, Grip-to-WT ratio and Grip-to-BMI ratio were negatively associated with the risk of developing high LDL-c, and only Grip-to-WT ratio was positively associated with the risk of developing IFG. Pinch strength and Pinch-to-MMP ratio were positively associated with the risk of developing high TG, low HDL-c, HBP and MetScore, and Pinch-to-MMP ratio was positively associated with the risk of developing high LDL-c. Pinch-to-WT ratio and Pinch-to-BMI ratio were negatively associated with the risk of developing high TG, low HDL-c, high LDL-c, HBP and MetScore, Pinch-to-WT ratio was negatively associated with the risk of developing IFG, and Pinch-to-MM ratio was negatively associated with the risk of developing low HDL-c.

**Figure 1:**
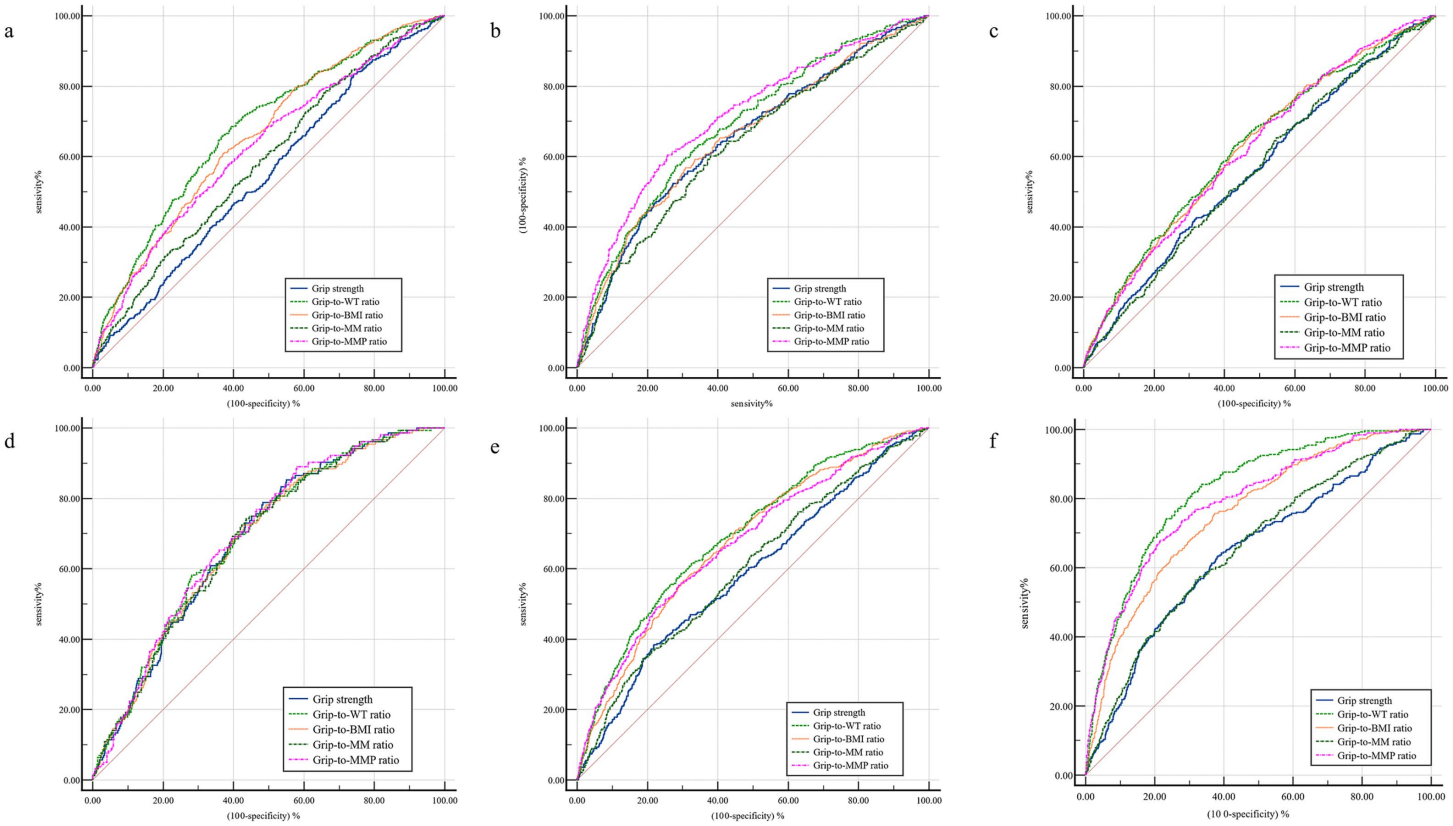
The ROC curves for predicting grip strength indices and abnormal cardiometabolic parameters. **(a)** High TG; **(b)** low HDL-c; **(c)** high HDL-c; **(d)** IFG; **(e)** HBP; **(f)** high MetScore. BMI, body mass index; HBP, high blood pressure; HDL-c, high-density lipoprotein cholesterol; IFG, impaired fasting glucose; LDL, low-density lipoprotein cholesterol; MetScore, clustered metabolic syndrome composite score; MM, muscle mass; MMP, muscle mass percentage; TG, triglycerides; WT, body weight.

**Figure 2:**
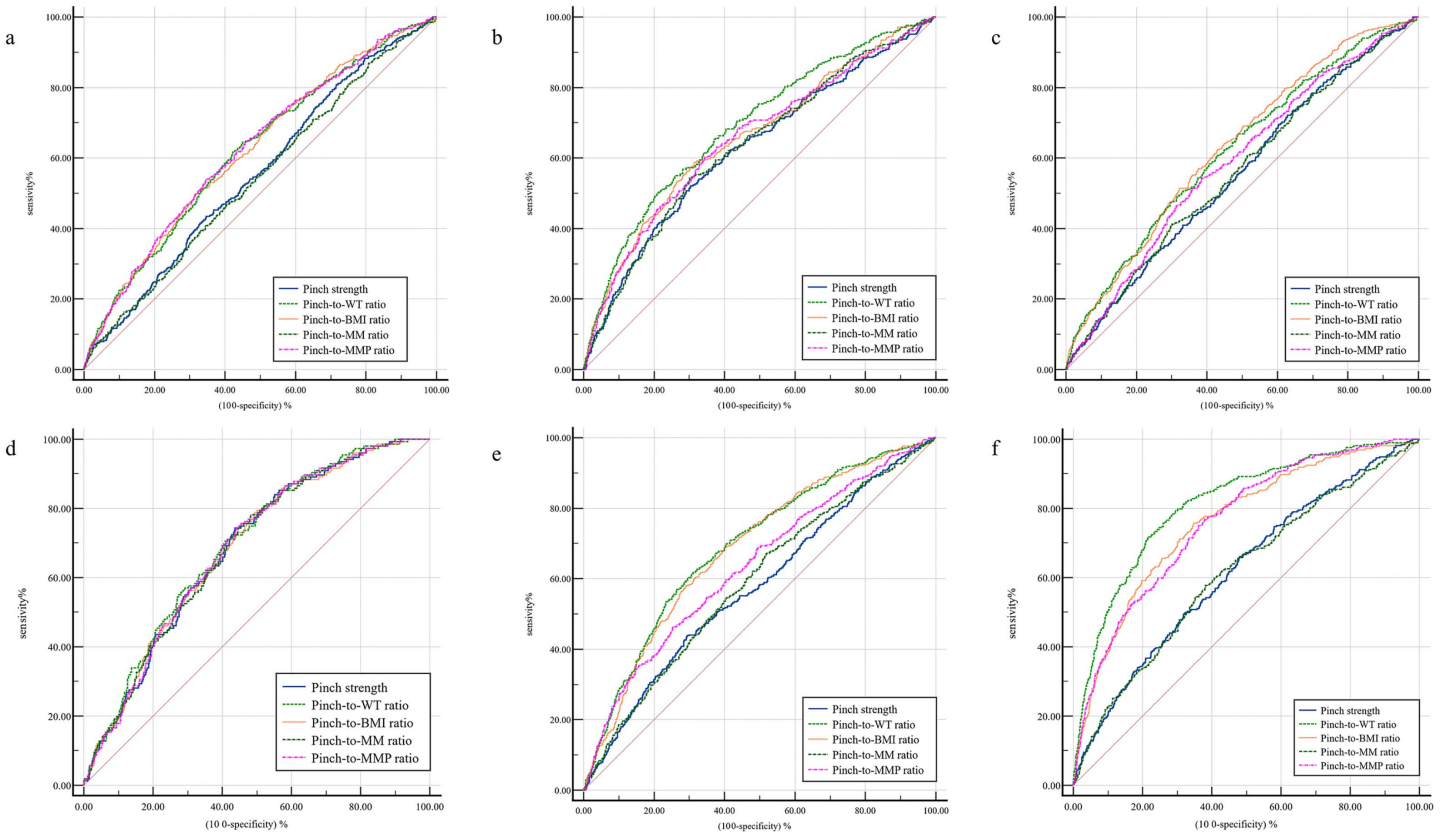
The ROC curves for predicting pinch strength indices and abnormal cardiometabolic parameters. **(a)** High TG; **(b)** low HDL-c; **(c)** high HDL-c; **(d)** IFG; **(e)** HBP; **(f)** high MetScore. BMI, body mass index; HBP, high blood pressure; HDL-c, high-density lipoprotein cholesterol; IFG, impaired fasting glucose; LDL, low-density lipoprotein cholesterol; MetScore, clustered metabolic syndrome composite score; MM, muscle mass; MMP, muscle mass percentage; TG, triglycerides; WT, body weight.

The ROC curves of each index of grip and pinch strength were shown in Figures 1, 2 and Tables 3, 4 present the relevant values of the ROC curves for the grip strength and pinch force indicators. Grip-to-WT ratio has the strongest ability to predict high LDL-c and MetScore, Grip-to-WT ratio and Grip-to-MMP ratio have stronger abilities to predict low HDL-c and HBP, and there is no significant difference between the two indicators (*p >* 0.05), Grip-to-WT ratio, grip-to-BMI ratio, and Grip-to-MMP ratio have stronger abilities to predict low HDL-c and there is no significant difference among the three indicators (*p >* 0.05). Pinch-to-WT ratio has the strongest ability to predict low HDL-c and MetScore, Pinch-to-WT ratio and Pinch-to-BMI ratio have stronger abilities to predict low HDL-c and HBP, and there is no significant difference between the two indicators (*p >* 0.05); Pinch-to-WT ratio, Pinch-to-BMI ratio and Pinch-to-BMI ratio have stronger abilities to predict high LDL-c, and there is no significant difference among the three indicators (*p >* 0.05). Overall, the Grip-to-WT ratio and the Pinch-to-WT ratio have stronger abilities to predict metabolic abnormalities.

**Table 3:** Analysis results of the ROC curve for predicting cardiovascular and metabolic risks based on grip strength index.

	ROC Performance		*p*-value for comparison				
	*AUC*	95%*CI*	*p* (vs Grip strength)	*p* (vs Grip-to-WT ratio)	*p* (vs Grip-to-BMI ratio)	*p* (vs Grip-to-MM ratio)	*p* (vs Grip-to-MMP ratio)
High TG							
Grip strength	0.548	(0.530, 0.565)		**<0.001**	**<0.001**	**0.036**	**<0.001**
Grip-to-WT ratio	0.679	(0.662, 0.695)	**<0.001**		**<0.001**	**<0.001**	**0.009**
Grip-to-BMI ratio	0.656	(0.639, 0.672)	**<0.001**	**<0.001**		**<0.001**	0.171
Grip-to-MM ratio	0.586	(0.568, 0.603)	**0.036**	**<0.001**	**<0.001**		0.061
Grip-to-MMP ratio	0.627	(0.610, 0.644)	**<0.001**	**0.009**	0.171	0.061	
Low HDL-c							
Grip strength	0.653	(0.636, 0.669)		0.078	0.843	0.198	**<0.001**
Grip-to-WT ratio	0.687	(0.671, 0.703)	0.078		**<0.001**	**<0.001**	0.188
Grip-to-BMI ratio	0.657	(0.640, 0.673)	0.843	**<0.001**		**0.001**	**0.004**
Grip-to-MM ratio	0.633	(0.616, 0.649)	0.198	**<0.001**	**0.001**		**<0.001**
Grip-to-MMP ratio	0.712	(0.696, 0.727)	**<0.001**	0.188	**0.004**	**<0.001**	
High LDL-c							
Grip strength	0.564	(0.547, 0.581)		**0.002**	**0.004**	0.758	**<0.001**
Grip-to-WT ratio	0.625	(0.608, 0.642)	**0.002**		0.876	**<0.001**	0.777
Grip-to-BMI ratio	0.624	(0.607, 0.641)	**0.004**	0.876		**<0.001**	0.824
Grip-to-MM ratio	0.560	(0.543, 0.577)	0.758	**<0.001**	**<0.001**		**<0.001**
Grip-to-MMP ratio	0.619	(0.603, 0.636)	**<0.001**	0.777	0.824	**<0.001**	
IFG							
Grip strength	0.685	(0.669, 0.701)		0.682	0.595	0.638	**0.023**
Grip-to-WT ratio	0.689	(0.672, 0.704)	0.682		0.154	0.236	0.488
Grip-to-BMI ratio	0.683	(0.666, 0.699)	0.595	0.154		0.898	0.077
Grip-to-MM ratio	0.683	(0.667, 0.699)	0.638	0.236	0.898		0.115
Grip-to-MMP ratio	0.694	(0.678, 0.710)	**0.023**	0.488	0.077	0.115	
HBP							
Grip strength	0.586	(0.569, 0.603)		**<0.001**	**<0.001**	0.464	**<0.001**
Grip-to-WT ratio	0.694	(0.678, 0.710)	**<0.001**		**0.001**	**<0.001**	0.266
Grip-to-BMI ratio	0.675	(0.658, 0.691)	**<0.001**	**0.001**		**<0.001**	0.900
Grip-to-MM ratio	0.600	(0.583, 0.617)	0.464	**<0.001**	**<0.001**		**<0.001**
Grip-to-MMP ratio	0.672	(0.656, 0.688)	**<0.001**	0.266	0.900	**<0.001**	
High MetScore							
Grip strength	0.645	(0.628, 0.661)		**<0.001**	**<0.001**	0.571	**<0.001**
Grip-to-WT ratio	0.821	(0.808, 0.834)	**<0.001**		**<0.001**	**<0.001**	0.080
Grip-to-BMI ratio	0.757	(0.742, 0.771)	**<0.001**	**<0.001**		**<0.001**	0.128
Grip-to-MM ratio	0.657	(0.640, 0.673)	0.571	**<0.001**	**<0.001**		**<0.001**
Grip-to-MMP ratio	0.789	(0.775, 0.803)	**<0.001**	0.080	0.128	**<0.001**	

Those highlighted in bold indicate statistical significance (bilateral *p* < 0.05, *p* values were from linear and logistics regression analysis).

**Table 4:** Analysis results of the ROC curve for predicting cardiovascular and metabolic risks based on pinch strength index.

	ROC Performance		*p*-value for comparison				
	*AUC*	95%*CI*	*p* (vs Pinch strength)	*p* (vs Pinch-to-WT ratio)	*p* (vs Pinch-to-BMI ratio)	*p* (vs Pinch-to-MM ratio)	*p* (vs Pinch-to-MMP ratio)
High TG							
Pinch strength	0.556	(0.539, 0.573)		**<0.001**	**<0.001**	**0.004**	**<0.001**
Pinch-to-WT ratio	0.620	(0.604, 0.637)	**<0.001**		0.817	**<0.001**	0.732
Pinch-to-BMI ratio	0.622	(0.605, 0.638)	**<0.001**	0.817		**<0.001**	0.807
Pinch-to-MM ratio	0.543	(0.526, 0.560)	**0.004**	**<0.001**	**<0.001**		**<0.001**
Pinch-to-MMP ratio	0.625	(0.608, 0.642)	**<0.001**	0.732	0.807	**<0.001**	
Low HDL-c							
Pinch strength	0.626	(0.609, 0.643)		**<0.001**	**0.032**	0.486	**<0.001**
Pinch-to-WT ratio	0.690	(0.674, 0.706)	**<0.001**		**<0.001**	**<0.001**	**0.024**
Pinch-to-BMI ratio	0.657	(0.640, 0.673)	**0.032**	**<0.001**		**0.003**	0.795
Pinch-to-MM ratio	0.633	(0.617, 0.650)	0.486	**<0.001**	**0.003**		0.088
Pinch-to-MMP ratio	0.653	(0.636, 0.669)	**<0.001**	**0.024**	0.795	0.088	
High *LDL-c*							
Pinch strength	0.556	(0.539, 0.574)		**<0.001**	**<0.001**	0.152	**<0.001**
Pinch-to-WT ratio	0.622	(0.605, 0.639)	**<0.001**		0.053	**<0.001**	**0.036**
Pinch-to-BMI ratio	0.633	(0.616, 0.649)	**<0.001**	0.053		**<0.001**	**0.008**
Pinch-to-MM ratio	0.562	(0.544, 0.579)	0.152	**<0.001**	**<0.001**		**0.002**
Pinch-to-MMP ratio	0.589	(0.572, 0.606)	**<0.001**	**0.036**	**0.008**	**0.002**	
IFG							
Pinch strength	0.683	(0.666, 0.699)		0.390	0.775	0.630	0.109
Pinch-to-WT ratio	0.692	(0.676, 0.708)	0.237		0.348	0.568	0.634
Pinch-to-BMI ratio	0.684	(0.667, 0.700)	0.775	0.090		0.718	0.668
Pinch-to-MM ratio	0.685	(0.668, 0.701)	0.630	0.148	0.718		0.905
Pinch-to-MMP ratio	0.685	(0.669, 0.701)	0.109	0.404	0.668	0.905	
HBP							
Pinch strength	0.576	(0.559, 0.593)		**<0.001**	**<0.001**	0.106	**<0.001**
Pinch-to-WT ratio	0.691	(0.675, 0.707)	**<0.001**		0.182	**<0.001**	**0.001**
Pinch-to-BMI ratio	0.683	(0.667, 0.699)	**<0.001**	0.182		**<0.001**	0.006
Pinch-to-MM ratio	0.589	(0.572, 0.606)	0.106	**<0.001**	**<0.001**		**<0.001**
Pinch-to-MMP ratio	0.636	(0.619, 0.653)	**<0.001**	**0.001**	0.006	**<0.001**	
High MetScore							
Pinch strength	0.619	(0.602, 0.636)		**<0.001**	**<0.001**	0.550	**<0.001**
Pinch-to-WT ratio	0.812	(0.798, 0.825)	**<0.001**		**<0.001**	**<0.001**	**0.002**
Pinch-to-BMI ratio	0.760	(0.745, 0.775)	**<0.001**	**<0.001**		**<0.001**	0.980
Pinch-to-MM ratio	0.614	(0.597, 0.631)	0.550	**<0.001**	**<0.001**		**<0.001**
Pinch-to-MMP ratio	0.761	(0.746, 0.775)	**<0.001**	**0.002**	0.980	**<0.001**	

Those highlighted in bold indicate statistical significance (bilateral *p* < 0.05, *p* values were from linear and logistics regression analysis).

## Discussion

In the present study, both the grip strength and pinch strength of children and adolescents aged 6–17 years tended to increase with age, and the overall grip strength and pinch strength were greater than those reported in previous research on Chinese children and adolescents (31, 32). The grip and pinch strengths of boys were greater than those of girls at all ages. The difference in muscle strength between boys and girls intensified after puberty (12 years of age and after), which is similar to the results of previous studies (33, 34), and is also consistent with the trend of changes in MM in Chinese children and adolescents: the hormonal changes in boys after puberty lead to an increase in testosterone (35), so that weight gain will be more dominated by the increase in nonadipose tissues (including skeletal muscle), while in girls after puberty, the increase in fat mass will be more pronounced (36), such leads to increased differences in muscle mass and strength between boys and girls after puberty.

The negative correlation of grip strength with cardiometabolic risk indicators seems to be well established in studies focusing on adults and older adults (37, 38). The correlation of pinch strength and cardiometabolic risk indicators has been found that pinch strength is significantly lower in diabetic patients (39). In the present study, grip strength and Grip-to-MMP ratio were positively associated with SBP, DBP and the risk of HBP but Grip-to-WT ratio, Grip-to-BMI ratio and Grip-to-MM ratio were negatively associated with SBP, DBP and the risk of HBP The association between muscle strength and blood pressure has always been controversial in previous studies, with more studies suggesting that muscle strength is negatively associated with blood pressure (40, 41), but some studies have also suggested that strong grip strength is associated with elevated blood pressure in adolescents (42) or has no relevance (43). Furthermore, a study indicates that the grip strength of school-aged children is positively correlated with blood pressure, and an increase in grip strength is associated with an elevated risk of hypertension in boys, but is not in girls (44).

Skeletal muscle is an important immune regulatory organ and generates myokines, which are a group of peptides and have anti-inflammatory and immunoprotective effects (45, 46). For instance, the irisin hormone produced by exercise can promote the transformation of white adipose tissue into brown adipose-like tissue, thereby increasing energy consumption, improving insulin sensitivity and glucose homeostasis (47). And muscle tissue is the primary site for glucose uptake and metabolism. Higher relative muscle strength usually indicates superior insulin sensitivity. Relative muscle weakness may lead to a decrease in the glucose disposal capacity of skeletal muscles, thereby exacerbating insulin resistance. In addition well-functioning muscles can secrete muscle hormones with anti-inflammatory effects (such as IL-6, which has anti-inflammatory properties after acute exercise), and they also help regulate pro-inflammatory factors derived from adipose tissue (such as TNF-*α*, IL-1β) (48). These might explain the observed association between relative grip strength and high TG, low HDL-cHBP, IFG and high MetScore in this study.

A Brazilian study in adolescents revealed that muscle strength values adjusted for MM were more representative of cardiometabolic risk in adolescents (49), but recent studies have suggested that the association between MM and metabolism is also affected by fat mass (50), meaning that high MM in high-fat children and adolescents does not imply reduced cardiometabolic risk. A study conducted in Shenzhen, China measured grip strength, and normalized relative grip strength by body mass index, and it was concluded that the low grip strength in the specific weight category and the relative grip strength classification were associated with higher z-scores of the cardiometabolic risk index compared to the classification based on gender-specific age references (51). Therefore, in this study, we analyzed the predictive ability of each grip strength indices on abnormal cardiometabolic parameters, and the results showed that the Grip/Pinch-to-WT ratio had the highest predictive value than all the other strength indices for cardiometabolic risk, and Grip/Pinch-to-MMP ratio and Grip/Pinch-to-BMI ratio are only second to Grip/Pinch-to-WT, possibly because the WT, MMP and BMI as indicators reflecting both MM and fat mass, can greatly eliminate the influence of different muscle and fat ratios on determining the association between grip strength and cardiometabolic risk. This is similar to the results of another cross-sectional study conducted in China (52).

The present study is a large sample survey about muscle strength covering almost the entire age group of children and adolescents. To our knowledge, it is the first report to systematically analyze the association between cardiometabolic risk and both absolute and normalized grip/pinch strength—especially those adjusted for body weight and composition—in Chinese children and adolescents. Unlike absolute strength measures, which are confounded by body size, these normalized indices more accurately reflect intrinsic muscle function and developmental quality, offering a purer metric of muscular health. Compared with screening for cardiometabolic risk by invasive blood test, body composition and muscle strength tests are easier to achieve and safer, our study provides new ideas and theoretical support for screening high cardiometabolic risk groups in children and adolescents. Our findings thus provide novel, functionally-grounded insights for identifying children and adolescents at high cardiometabolic risk. Furthermore, the present study compared the predictive ability of grip and pinch strength indices for abnormal cardiometabolic parameters through ROC curves and explored the best predictor of cardiometabolic risk.

### Limitations

There are some limitations in this study. First, the dominant hand and the order of each hand’s measurements of children and adolescents were not recorded in this study, which may have had some influence on the measured values of grip and pinch strengths, but we minimized the above measurement bias in our study by retaining the maximum grip and pinch strength values of both hands. Second, due to the large number of cardiometabolic risk-related factors, only the age, sex, annual household income, dairy products, vegetable, fruits, fried food, sugared beverage and daily exercise duration, and grip strength of the children were taken into account in this study, and other related factors such as the impact of children’s puberty development cannot be ruled out. Which possibly explained the AUCs were all less than 0.85 in this study. Finally, the data for the current study are from a baseline survey in Beijing, the extrapolation was limited and causal associations could not be inferred. Therefore, it is necessary to collect as many factors as possible affecting cardiometabolic risk indicators during future cohort follow-ups in the future and to further determine the causal associations between muscle strength and cardiometabolic risk factors.

## Conclusion

The Grip-to-WT ratio emerged as a particularly robust predictor for most cardiometabolic risk parameters, including the composite MetScore. Incorporating simple measures like the Grip/Pinch-to-WT ratio into health assessments could enhance the early identification of youths at risk for cardiometabolic diseases.

## Data Availability

The original contributions presented in the study are included in the article/Supplementary material, further inquiries can be directed to the corresponding author.
