# Supplementary material for: Grip and pinch strengths and its association with cardiometabolic risk in children and adolescents aged 6 to 17 years
Source: Front Nutr. 2026 Feb 17;13:1763759. doi: 10.3389/fnut.2026.1763759 (PMC12953518; doi:10.3389/fnut.2026.1763759)
Supplement: Supplementary file 1 [file Table_1.DOCX]

**Online supplementary material**

| **Table S1. Grip and pinch strengths of children and adolescents stratified by sex and age (n=3252)** | | | | | | | | |  |
| --- | --- | --- | --- | --- | --- | --- | --- | --- | --- |
| **SEX** | **AGE(age)** | **N** | **Grip strength(kg)** | | |  | **Pinch strength(kg)** | | |
|  |  |  | ***M*** | ***P_25_*** | ***P_75_*** |  | ***M*** | ***P_25_*** | ***P_75_*** |
| **Boys** | 6 | 151 | 8.8 | 7.4 | 10.2 |  | 3.0 | 2.5 | 3.4 |
|  | 7 | 125 | 10.8 | 9.6 | 12.5 |  | 3.3 | 3.0 | 3.8 |
|  | 8 | 114 | 12.7 | 10.5 | 14.8 |  | 3.6 | 3.2 | 4.2 |
|  | 9 | 99 | 15.4 | 12.5 | 17.7 |  | 4.5 | 3.7 | 4.9 |
|  | 10 | 114 | 17.5 | 14.7 | 19.8 |  | 4.7 | 4.0 | 5.3 |
|  | 11 | 87 | 18.7 | 16.5 | 22.4 |  | 5.1 | 4.4 | 5.9 |
|  | 12 | 208 | 27.2 | 21.5 | 32.1 |  | 6.6 | 5.5 | 7.7 |
|  | 13 | 234 | 30.9 | 25.9 | 36.0 |  | 7.2 | 6.1 | 8.4 |
|  | 14 | 194 | 36.6 | 31.7 | 42.1 |  | 8.3 | 6.9 | 9.6 |
|  | 15 | 114 | 39.4 | 33.4 | 45.1 |  | 8.7 | 7.5 | 10.0 |
|  | 16 | 80 | 43.5 | 36.7 | 50.6 |  | 8.6 | 7.8 | 10.0 |
|  | 17 | 90 | 45.2 | 37.5 | 51.3 |  | 9.6 | 8.3 | 10.6 |
|  | ALL | 1610 | 24.2 | 13.8 | 35.9 |  | 6.1 | 4.0 | 8.2 |
| **Girls** | 6 | 143 | **8.4** | **6.9** | **9.8** |  | **2.7** | **2.3** | **3.1** |
|  | 7 | 98 | **9.6** | **8.3** | **10.8** |  | **3.1** | **2.8** | **3.6** |
|  | 8 | 110 | **11.5** | **9.6** | **13.4** |  | **3.4** | **2.9** | **4.0** |
|  | 9 | 95 | **13.6** | **11.5** | **16.4** |  | **3.9** | **3.3** | **4.8** |
|  | 10 | 108 | **16.0** | **13.2** | **19.2** |  | **4.4** | **3.8** | **5.1** |
|  | 11 | 93 | **19.2** | **16.2** | **22.0** |  | **5.1** | **4.2** | **6.0** |
|  | 12 | 224 | **23.5** | **20.3** | **26.7** |  | **5.8** | **5.2** | **6.7** |
|  | 13 | 232 | **23.1** | **20.4** | **26.5** |  | **6.2** | **5.4** | **7.0** |
|  | 14 | 205 | **24.5** | **21.8** | **27.4** |  | **6.2** | **5.5** | **7.0** |
|  | 15 | 147 | **26.0** | **23.2** | **29.5** |  | **6.5** | **5.6** | **7.5** |
|  | 16 | 99 | **29.2** | **25.3** | **33.2** |  | **6.6** | **5.7** | **7.7** |
|  | 17 | 88 | **27.9** | **23.2** | **32.0** |  | **6.5** | **5.7** | **7.5** |
|  | ALL | 1642 | **21.1** | **13.5** | **26.1** |  | **5.4** | **3.9** | **6.6** |
| Differences of the grip or pinch strengths between sex were analyzed using rank sum tests due to not normally distributed. Those highlighted in bold indicate statistical significance (bilateral *P* < 0.05).   \| **Table S2. Characteristics of participants with complete data for all outcomes investigated in this study (n=3252)** \| \| \| \| \| \| \| --- \| --- \| --- \| --- \| --- \| --- \| \| **Variable** \| **Boys** \| **Girls** \| **All** \| ***t / Z / x^2^*** \| ***P*** \| \| **1610 (49.51)** \| **1642 (50.49)** \| **3252** \| \| *WT (cm)* \| 53.5±22.9 \| 47.9±17.0 \| 50.7±20.4 \| **7.886** \| **<0.001** \| \| *MM (kg)* \| 38.3±14.5 \| 31.65±8.5 \| 35.0±12.3 \| **15.990** \| **<0.001** \| \| *MMP (%)* \| 73.6±9.9 \| 68.4±8.5 \| 71.0±9.6 \| **16.102** \| **<0.001** \| \| *BMI (kg/m^2^)* \| 21.3±5.4 \| 20.3±4.8 \| 20.8±5.1 \| **5.307** \| **<0.001** \| \| *TG (mmol·L^-1^)* \| 0.79 [0.59, 1.10] \| 0.86 [0.65, 1.12] \| 0.83 [0.62, 1.11] \| **-2.628** \| **0.009** \| \| *HDL-c (mmol·L^-1^)* \| 1.40±0.33 \| 1.44±0.31 \| 1.42±0.32 \| **-4.315** \| **<0.001** \| \| *LDL-c (mmol·L^-1^)* \| 2.56±0.75 \| 2.60±0.71 \| 2.58±0.73 \| -1.746 \| 0.081 \| \| *FPG (mmol·L^-1^)* \| 5.01±0.7 \| 4.89±0.43 \| 4.95±0.58 \| **6.284** \| **<0.001** \| \| *SBP (mmHg)* \| 114.55±12.40 \| 109.18±10.82 \| 111.84±11.94 \| **13.167** \| **<0.001** \| \| *DBP (mmHg)* \| 61.81±7.33 \| 61.92±7.42 \| 61.87±7.37 \| -0.426 \| 0.670 \| \| *MetScore* \| 0.01±0.61 \| 0.03±0.60 \| 0.02±0.61 \| -0.939 \| 0.348 \| \| **Abnormal cardiometabolic parameters** \| \|  \|  \|  \|  \| \| *HBP* \| 232 (14.41) \| 195 (11.88) \| 427 (13.13) \| **4.577** \| **0.032** \| \| *high TG* \| 220 (13.66) \| 206 (12.55) \| 426 (13.10) \| 0.894 \| 0.344 \| \| *low HDL-c* \| 187 (11.61) \| 125 (7.61) \| 312 (9.59) \| **15.013** \| **<0.001** \| \| *high LDL-c* \| 214 (13.29) \| 209 (12.73) \| 423 (13.01) \| 0.228 \| 0.633 \| \| *IFG* \| 97 (6.02) \| 58 (3.53) \| 155 (4.77) \| **11.127** \| **0.001** \| \| *high MetScore* \| 259 (16.09) \| 266 (16.20) \| 525 (16.14) \| 0.008 \| 0.930 \| \| Note: MetScore = (zBMI+ zFPG + zlgTG - zHDL-c + zSBP+ zDBP)/6.  *P* values were from t tests, rank sum tests and chi-square tests. Those highlighted in bold indicate statistical significance (bilateral *P* < 0.05).  Abbreviations: BMI, body mass index; DBP, diastolic blood pressure; FPG, fasting blood glucose; HBP, high blood pressure. HDL-c, high-density lipoprotein cholesterol; IFG, Impaired fasting glucose; LDL, low-density lipoprotein cholesterol; MetScore, clustered metabolic syndrome composite score; MM, muscle mass; MMP, muscle mass percentage; SBP, systolic blood pressure; TG, triglycerides; WT, body weight. \| \| \| \| \| \|   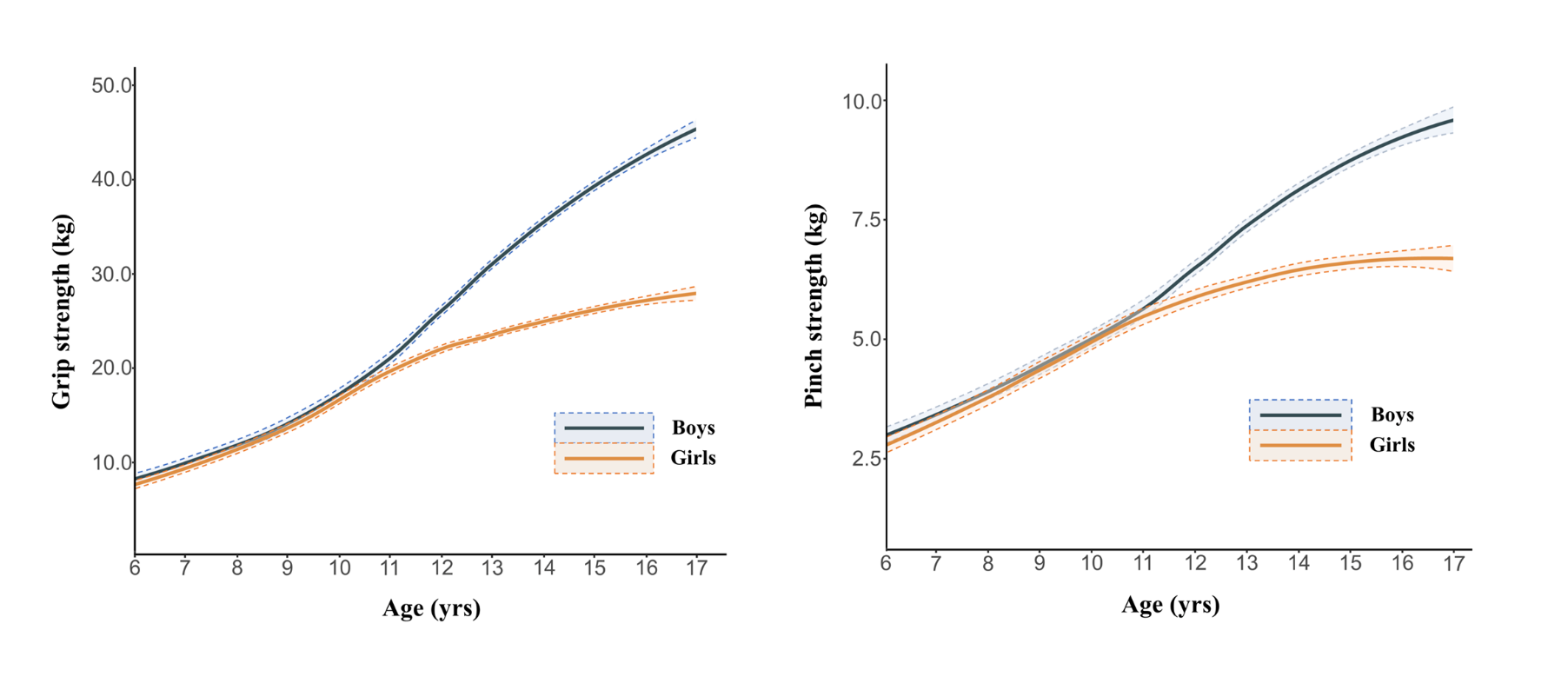  **Figure S1.** The trends of changes in grip and pinch strength with age in children and adolescents stratified by sex. | | | | | | | | | |
