# Supplementary material for: Grip and pinch strengths and its association with cardiometabolic risk in children and adolescents aged 6 to 17 years
Source: Front Nutr. 2026 Feb 17;13:1763759. doi: 10.3389/fnut.2026.1763759 (PMC12953518; doi:10.3389/fnut.2026.1763759)
Supplement: Supplementary file 3 [file Table_3.DOCX]

**Abbreviations:**

AUCs: areas under the curve

BMI: body mass index

DBP: diastolic blood pressure

FPG: fasting blood glucose

HDL-c: high-density lipoprotein cholesterol

IFG: impaired fasting glucose

LDL-c: low-density lipoprotein cholesterol

MetScore: Clustered metabolic syndrome composite score

MM: muscle mass

MMP: muscle mass percentage

ROC: the receiver operating characteristic

SBP: systolic blood pressure

STROBE: strengthening the reporting of observational studies in epidemiology

TG: triglycerides

WT: body weight
